# Phenomics and transcriptomics analyses reveal deposition of suberin and lignin in the short fiber cell walls produced from a wild cotton species and two mutants

**DOI:** 10.1371/journal.pone.0282799

**Published:** 2023-03-09

**Authors:** Hee Jin Kim, Yongliang Liu, Gregory N. Thyssen, Marina Naoumkina, James Frelichowski

**Affiliations:** 1 USDA-ARS, Southern Regional Research Center, Cotton Fiber Bioscience Research Unit, New Orleans, LA, United States of America; 2 USDA-ARS, Southern Regional Research Center, Cotton Structure and Quality Research Unit, New Orleans, LA, United States of America; 3 USDA-ARS-SPARC, Crop Germplasm Research Unit, College Station, TX, United States of America; University of Sao Paulo, BRAZIL

## Abstract

Fiber length is one of the major properties determining the quality and commercial value of cotton. To understand the mechanisms regulating fiber length, genetic variations of cotton species and mutants producing short fibers have been compared with cultivated cottons generating long and normal fibers. However, their phenomic variation other than fiber length has not been well characterized. Therefore, we compared physical and chemical properties of the short fibers with the long fibers. Fiber characteristics were compared in two sets: 1) wild diploid *Gossypium raimondii* Ulbrich (short fibers) with cultivated diploid *G*. *arboreum* L and tetraploid *G*. *hirsutum* L. (long fibers); 2) *G*. *hirsutum* short fiber mutants, Ligon-lintless 1 (*Li*_*1*_) and 2 *(Li*_*2*_) with their near isogenic line (NIL), DP-5690 (long fibers). Chemical analyses showed that the short fibers commonly consisted of greater non-cellulosic components, including lignin and suberin, than the long fibers. Transcriptomic analyses also identified up-regulation of the genes related to suberin and lignin biosynthesis in the short fibers. Our results may provide insight on how high levels of suberin and lignin in cell walls can affect cotton fiber length. The approaches combining phenomic and transcriptomic analyses of multiple sets of cotton fibers sharing a common phenotype would facilitate identifying genes and common pathways that significantly influence cotton fiber properties.

## Introduction

Cotton (*Gossypium sp*.) is the most economically important natural fiber in the world [1]. In addition to the agronomic importance, cotton fibers are also utilized as an ideal biological model for studying molecular mechanisms involved in cell elongation and cell wall biogenesis because cotton fiber cells are unicellular and larger and longer than any other plant cell [2]. Cotton fiber development is divided into four overlapping stages: 1) initiation, 2) primary cell wall (PCW) biosynthesis characterized by fiber elongation, 3) secondary cell wall (SCW) biosynthesis characterized by cellulose production, and 4) maturation process [3]. The overlapping period between PCW and SCW stages is often classified as a transition stage due to its importance for fiber cell wall biosynthesis. Genotypes and growth conditions greatly affect the period of each developmental stage. Cytochemical analyses showed that cultivated *G*. *hirsutum* fibers exhibit thickened SCW consisting of nearly pure cellulose [4, 5]. Chemical analyses detected very low levels (0.4–1% of dried weight) of lignin-like phenolics from cultivated *G*. *hirsutum* fibers [6].

Fiber length is a major physical property determining fiber quality [7]. Wild diploid *G*. *raimondii* (D_5_ genome) produces short fibers that are agronomically inferior to cultivated diploid *G*. *arboreum* (A_2_ genome) as well as cultivated polyploid *G*. *hirsutum* (AD_1_ genome) [8]. Among the wild diploid cotton species, *G*. *raimondii* is considered as the closest extant relative to the D_T_ subgenome of the most widely cultivated polyploid *G*. *hirsutum* (AD_1_ genome) [9]. It also shares cytological, morphological, and phenogenetical similarities to the polyploid cotton species. Thus, the D_5_ genome of wild diploid *G*. *raimondii* was the first sequenced among cotton species [10–12]. *G*. *arboreum* is suggested to be the closest extant relative of the A_T_ subgenome of the polyploid cotton species [9]. The A_2_ genome of cultivated diploid *G*. *arboreum* SXY1 was sequenced [13]. The AD_1_ genome was first sequenced from cultivated polyploid *G*. *hirsutum* TM-1 [14, 15]. Those cotton genome sequences with their transcriptomic data have been available in public databases [16], and extensively utilized to study natural diversity and evolution of cotton species as well as cotton genetic research.

In polyploid *G*. *hirsutum*, two short fiber mutants, Ligon-lintless 1 (*Li*_*1*_) [17] and Ligon-lintless 2 (*Li*_*2*_) [18] have been reported. *Li*_*1*_ mutant shows pleiotropic effects on both fiber and non-fiber tissues, whereas *Li*_*2*_ mutant phenotype is specifically detected in fiber tissue. *G*. *hirsutum* short fiber mutant plants containing *Li*_*1*_ [19] or *Li*_*2*_ [20] gene were crossed with *G*. *hirsutum* DP-5690 plants producing long fibers. F_1_ progeny were backcrossed for five generations (BC_5_) by single seed decent (SSD) to DP-5690 which served as the recurrent parent in each backcross. The two short fiber mutants with their NIL, DP-5690 have been used as a model system to study fiber elongation [21–25]. Our group has shown that mutations in the *actin* [25] and in the putative *Ran Binding Protein 1* involved in nucleocytoplasmic transport [23] are responsible for the short fiber phenotypes of *Li*_*1*_ and *Li*_*2*_ mutants, respectively.

**Fig 1:**
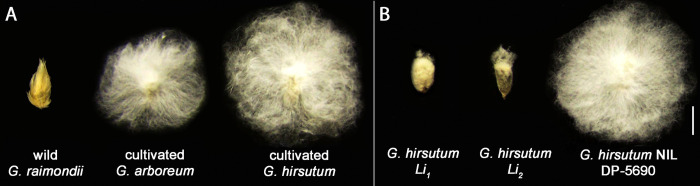
Morphology of two sets of cottonseeds for an integrative analysis. A. The 1^st^ set is composed of three cotton species including wild diploid *G*. *raimondii* D_5_-31 (D_5_ genome), cultivated diploid *G*. *arboreum* A_2_-100 (A_2_ genome), and cultivated polyploid *G*. *hirsutum* TM-1 (AD_1_ genome). B. The 2^nd^ set consists of *G*. *hirsutum Li*_*1*_ and *Li*_*2*_ mutants producing short fibers along with their near isogenic line (NIL) DP-5690 producing long fibers. The single seeds with associating cotton fibers were harvested from the open bolls, combed and photographed. The bars represent 1 cm in length.

The short fiber phenotypes are commonly observed in polyploid *G*. *hirsutum Li*_*1*_ and *Li*_*2*_ mutants and wild diploid *G*. *raimondii* (Fig 1). However, it is not known if the short fibers share any common mechanisms to impair fiber elongation. In this study, we determined and compared the chemical compositions and transcriptomic profiles of two sets of cotton fibers. The first set (Fig 1A) consisted of three cotton species, wild diploid *G*. *raimondii* (short fibers), diploid cultivated *G*. *arboreum* (long fibers) and polyploid cultivated *G*. *hirsutum* (long fibers). The second set (Fig 1B) was composed of *G*. *hirsutum Li*_*1*_ (short fibers), *Li*_*2*_ (short fibers), and their NIL, *G*. *hirsutum* DP-5690 (long fibers). Integrations of chemical properties and transcriptomic profiles of the two sets showed that suberin and lignin are commonly associated with the short fiber phenotypes.

## Materials and methods

### Plant materials and growth conditions

This research was approved by the biosafety committee of USDA-ARS-SRRC. Field research was performed according to the policy and practices of USDA-ARS. The cottonseeds with Plant Introduction (PI) or Plant Variety Protection (PVP) numbers including diploid *G*. *arboreum* A_2_-100 (PI 529728), and *G*. *raimondii* D_5_-6 (PI530903) and D_5_-31 (PI 530928) as well as polyploid *G*. *hirsutum* Texas Marker 1 (TM-1, PI 607172), Sure-Grow 747 (SG-747, PVP 9800118) and Delta Pine 5690 (DP-5690, PVP 9100116) were obtained from the U.S. National Cotton Germplasm Collection (NCGC). The cottonseeds of short fiber mutants, Ligon-lintless 1 (*Li*_*1*_) mutant, Ligon-lintless 2 (*Li*_*2*_), and diploid *G*. *arboreum* Shixiya1 (SXY1) were provided by Dr. Rickie Turley of USDA-ARS-SEA and Dr. Xianliang Song of Shandong Agricultural University, China.

Each variety of *G*. *arboreum* (A_2_-100 and SXY1) and *G*. *hirsutum* (TM-1, SG-747, DP-5690, *Li*_*1*_, and *Li*_*2*_) were planted on two-row plots located at the Southern Regional Research Center (New Orleans, LA; 2017) with naturally neutral-day conditions. The soil type of the cotton plot was aquents dredged over alluvium in an elevated location to provide adequate drainage. Single row plots were 12 m long with approximately 40 plants per plot. The distance between two rows was 0.5 m, and the distance between two plants within a row was 0.3 m. To minimize environmental effects, boll samples were not collected from plants on the perimeter of the field and the end of each row. At harvest, approximately 60 naturally opened bolls were randomly collected from two plots for each cotton variety, and separated into two biological replicates with 30 bolls per biological replicate for further analyzing physical and chemical properties of each cotton variety.

To collect developing fibers at various developmental stages, wild diploid *G*. *raimondii* D_5_-6 and D_5_-31 along with *G*. *arboreum* and *G*. *hirsutum* were grown in a growth chamber (Percival Intellus Environmental Controller, Perry, IA) in 8 L pots at 28°C (day) / 24°C (night) with a short photoperiod condition (9h day light, 300 μmolm^-2^ s^-1^) during the vegetative stage, and reduced to 26°C (day)/ 18°C (night) during flowering and boll development stages. The pots were filled with Metro-Mix 350 soil. For fiber length measurement, two plants of wild diploid *G*. *raimondii* D_5_-6 were grown in 167 L containers at an NCGC greenhouse located at College Station, Texas during a winter season for a short photoperiod condition. The two *G*. *raimondii* D_5_-6 plants produced four bolls, and separated into two biological replicates with two bolls per biological replicate. To obtain sufficient *G*. *raimondii* fibers for fiber length and chemical analyses, *G*. *raimondii* D_5_-31 was grown perennially at the cotton winter nursery at Tecoman, Colima, Mexico in association with the location of the Instituto Nacional de Investigaciones Forestales, Agrícolas y Pecuarias [26]. Three *G*. *raimondii* D_5_-31 plants (240 days after planting) were transplanted on the ground of the cotton winter nursery. In the second year, they produced 400 bolls that were separated into two biological replicates with 200 bolls per biological replicate for further analyzing fiber length and chemical analyses. All *G*. *raimondii* grown in the growth chamber, greenhouse, and cotton winter nursery produced a common phenotype demonstrating short and green colored fibers.

### Cotton fiber length measurements

Maximum fiber lengths were estimated by placing ovules on a watch-glass and gently spraying fibers with a stream of distilled water as described by Schubert et al. [27]. Ten to thirty cotton bolls were randomly selected from each biological replicate samples of *G*. *arboreum* (A_2_-100 and SXY1), *G*. *raimondii* (D_5_-6 and D_5_-31) and *G*. *hirsutum* (TM-1, SG-747, DP-5690, *Li*_*1*_, and *Li*_*2*_). Single cotton seeds were randomly selected from an individual cotton boll. The distance between the chalazal end of the selected seeds and the tip of the spread fibers were measured to the nearest 0.1 mm with a digital caliper. Mean maximum fiber length of each cotton variety was obtained by measuring the randomly selected seeds from two biological replicates.

### Updegraff cellulose assay

Cellulose contents of developed cotton fibers were measured by the modified Updegraff method [28]. Five cotton bolls were randomly selected from each biological replicate samples of the cultivated *G*. *arboreum* (A_2_-100 and SXY1) and *G*. *hirsutum* (TM-1, SG-747, and DP-5690) producing long fibers. Two to six cotton bolls were also randomly selected from each biological replicate samples of the *G*. *raimondii* (D_5_-6 and D_5_-31) and *G*. *hirsutum* mutants (*Li*_*1*_ and *Li*_*2*_) generating short fibers. Dried fiber samples of the selected bolls were manually harvested, and cut into small pieces. Ten milligrams of the blended fibers were placed in 5 mL Reacti-Vials^TM^ (Thermol Fisher Scientific, Waltham, MA), and hydrolyzed with acetic-nitric reagent (a mixture of 73% acetic acid, 9% nitric acid and 18% water). The remaining cellulose was hydrolyzed with 67% sulfuric acid (v/v) and measured by a colorimetric assay with anthrone with Avicel PH-101 (FMC, Rockland, ME, USA) as a cellulose standard. Mean cellulose content of each cotton variety was obtained by measuring the randomly selected cotton bolls from two biological replicates.

### Attenuated Total Reflection Fourier Transform Infrared (ATR FT-IR) spectral collection and data analysis

Five cotton bolls were randomly selected from each biological replicate samples of cultivated *G*. *arboreum* A_2_-100 and *G*. *hirsutum* TM-1 and DP-5690 producing long fibers as well as wild *G*. *raimondii* D_5_-31 and *G*. *hirsutum Li*_*1*_ and *Li*_*2*_ mutants generating short fibers. Dried fiber samples were manually harvested from the selected bolls, and divided into six portions that were directly scanned without further processing. Average spectra of each replicate samples were obtained from the spectra from the six portions of the sample. Mean spectra of each cotton variety were obtained by measuring the randomly selected cotton bolls from two biological replicates. All samples were scanned by an FTS 3000MX FT-IR spectrometer (Varian Instruments, Randolph, MA, USA) equipped with a ceramic source, KBr beam splitter, and deuterated triglycine sulfate (DTGS) detector and attenuated total reflection (ATR) attachment according to the methods that were previously described in Liu and Kim [29]. The spectra were normalized by dividing the intensity of an individual band in the 1800–600 cm^-1^ region with the average intensity in that 1800–600 cm^-1^ region, and subsequent principal component analysis (PCA) characterization was performed in the 3000–1200 cm^-1^ IR region with mean centering (MC), multiplicative scatter correction (MSC), and Savitzky–Golay first-derivative (13 points) spectral pretreatment and with leave-one-out cross-validation method.

### Pyrolysis-molecular beam mass spectrometry lignin analysis

Five cotton bolls were randomly selected from each replicate samples of the cultivated *G*. *arboreum* A_2_-100 and *G*. *hirsutum* TM-1 as well as wild *G*. *raimondii* D_5_-31. Two replicated samples of the dried cotton fibers of *G*. *raimondii* D_5_-31, *G*. *arboreum* A_2_-100, and *G*. *hirsutum* TM-1 were cut in a Wiley mill into 20 mesh. Average contents of each cotton variety were obtained by measuring the randomly selected cotton bolls from two biological replicates. Lignin analysis was performed with pyrolysis molecular beam mass spectroscopy (pr-MBMS) by Complex Carbohydrate Research Center (CCRC) at University of Georgia. Duplicated cotton samples along with control samples including NIST 8492 (lignin content, 26.2%) and aspen standards were pyrolyzed at 500°C and the volatile compounds were analyzed for lignin using a molecular beam mass spectrometer (Extrel Core Mass Spectrometers). The raw data were processed through UnscramblerX 10.1 software to obtain the principal components and raw lignin data. *G*. *arboreum* A_2_-100 fibers exclusively composed of cellulose (95.6~100%) with the lowest lignin level among cotton species was also used as the lignin base line for all tested cotton samples.

### Transcriptomic analyses

RNA-seq reads for the cotton materials shown in Table 1 were retrieved from the NCBI SRA database. These reads were aligned to the JGI *G*. *raimondii* reference genome [10] using gsnap software and reads that mapped to annotated genes were counted using bedtools software [30, 31]. RNA-seq expression analysis was conducted following the PolyCat pipeline as previously described [24, 32]. Briefly, all reads were aligned to the JGI *G*. *raimondii* reference genome, then the PolyCat software assigned each categorizable read to either the A_T_ or D_T_ subgenome based on an index of homeoSNPs. Using the retrieved RNA-seq reads (Table 1 and S1 Table), RPKM (reads per kilobase of transcript per million reads mapped) numbers were determined, and specifically or differentially expressed genes in developing D_5_, *Li*_*1*_ or *Li*_*2*_ fibers at elongating PCW or wall-thickening SCW stage were identified and annotated based on the best hit by BLAST search with The *Arabidopsis* Information Resource version 10 (TAIR 10). The GO enrichment analysis was performed using agriGO v2.0 Singular Enrichment Analysis [33].

**Table 1:** Database of RNA-seq used in this study.

Cotton species (genome)	Fiber stages (DPA)	NCBI SRA RNA-seq Database	Reference
*G*. *raimondii* (D_5_)	PCW (10)	SRX062246	[34]
	SCW (20)	SRX062252	
*G*. *arboreum* (A_2_)	PCW (10)	SRX062247	
	SCW (20)	SRX062251	
*G*. *hirsutum* TM-1 (AD_1_)	PCW (10)	SRX202895	[35]
	PCW (20)	SRX202896	
	SCW (28)	SRX15944380	[36]
*G*. *hirsutum* DP-5690 (AD_1_)	PCW (8)	SRX853935, SRX853937, SRX 853939	[24]
*G*. *hirsutum Li*_*1*_ in DP-5690 background (AD_1_)	PCW (8)	SRX853916, SRX853922, SRX853932,	
*G*. *hirsutum Li*_*2*_ in DP-5690 background (AD_1_)	PCW (8)	SRX853891, SRX853905, SRX 853912	

### Image and statistical analyses

Image composites were constructed using Adobe Photoshop 2022 software. Statistical analyses and construction of graphs were performed using t-test and Prism version 7.05 software (Graph-Pad Software, Inc., San Diego, CA). The p value cutoff for significance was 0.05 with four levels at <0.05 (*), <0.01 (**), <0.001 (***), and < 0.0001 (****).

## Results

### Phenotypic characterizations of cotton fibers

**Fig 2:**
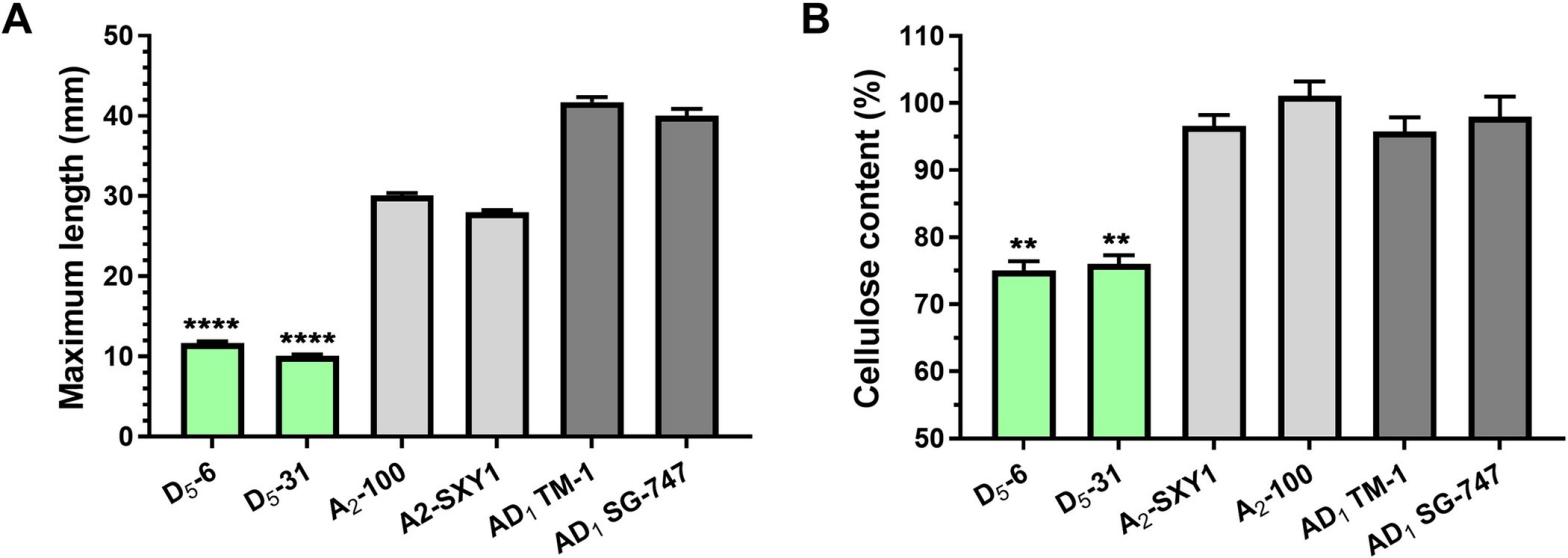
Fiber phenotypic variations between wild diploid *G*. *raimondii* and two cultivated cotton species (1^st^ set). A. Maximum fiber length. Fiber length was manually measured from cottonseeds of wild *G*. *raimondii* (D_5_-6 and 31), cultivated *G*. *arboreum* (A_2_-100 and SXY1), and cultivated *G*. *hirsutum* (TM-1 and SG-747). B. Cellulose content. The error bars represent standard deviation. **p value < 0.01; ****p value *<*0.0001.

**Fiber phenotypic characterizations of wild diploid *G*. *raimondii* and two cultivated cotton species (1**^**st**^ **set).** There were notable phenotypic variations of cottonseeds among three different cotton species including a wild diploid *G*. *raimondii*, cultivated diploid *G*. *arboreum*, and cultivated allotetraploid *G*. *hirsutum* (1^st^ set, Fig 1A). The average maximum fiber lengths of diploid *G*. *raimondii* D_5_-6 (11.7 mm) and D_5_-31 (10.1 mm) were significantly (p<0.0001) shorter than the two diploid of *G*. *arboreum* A_2_-100 (30.1 mm) and SXY1 (28.0 mm) as well as the two polyploid *G*. *hirsutum* TM-1 (41.7 mm) and SG-747 (40.1 mm) (Fig 2A). Similarly, the cellulose contents of the two *G*. *raimondii* fibers (75.0~78.0%) were significantly (p<0.01) less than those of the two *G*. *arboreum* (95.6~100%) and the two *G*. *hirsutum* (95.8~98.0%) fibers that were almost exclusively composed of cellulose (Fig 2B).

#### Fiber phenotypic variations of short fiber *G*. *hirsutum* mutants and their near isogenic line DP-5690 (2^nd^ set)

**Fig 3:**
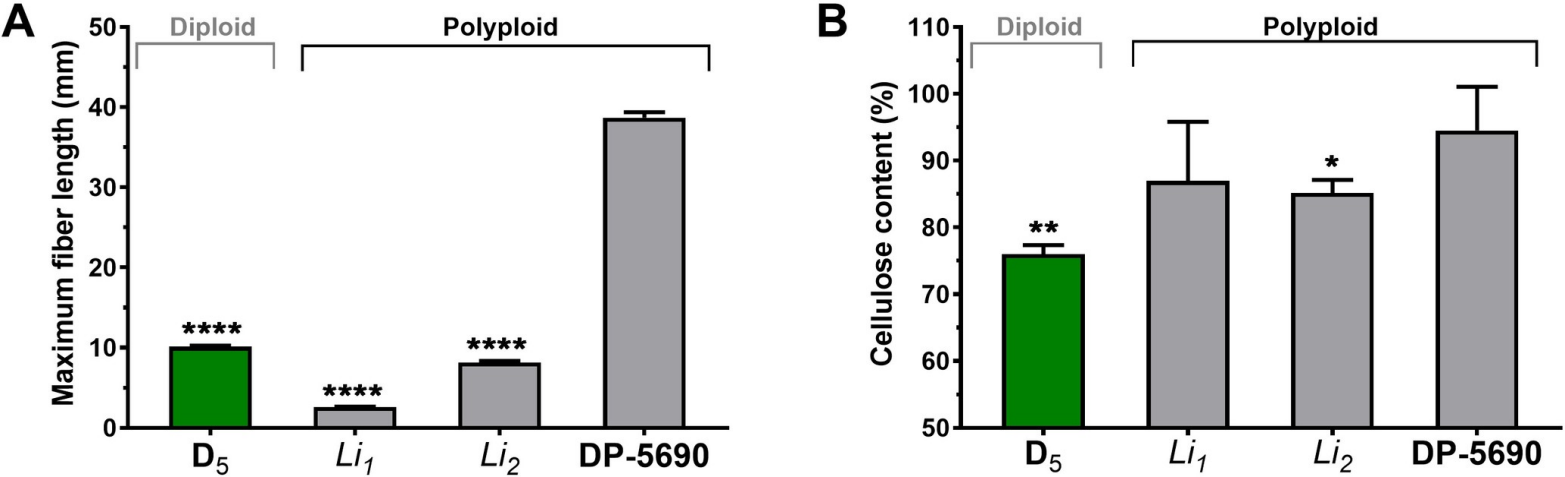
Fiber phenotype variations between short fiber *G*. *hirsutum* mutants and their near isogenic line DP-5690 (2^nd^ set). A. Maximum fiber length. Fiber length of *G*. *hirsutum* short fiber *Li*_*1*_ and *Li*_*2*_ mutants were manually measured and compared with those of their NIL, *G*. *hirsutum* DP-5690 and wild diploid *G*. *raimondii* D_5_-31 (D_5_). B. Cellulose content. The error bars represent standard deviation. *p value < 0.05; **p value < 0.01; ****p value *<*0.0001.

The fiber lengths and cellulose content of *G*. *hirsutum Li*_*1*_ and *Li*_*2*_ mutants were also compared with their NIL cotton, DP-5690 (2^nd^ set, Fig 1B). Average maximum fiber lengths of *G*. *hirsutum Li*_*1*_ (2.6 mm) and *Li*_*2*_ (8.1 mm) mutants were significantly (p<0.0001) shorter than their NIL, DP-5690 (38.7 mm) (Fig 3A). Average cellulose content of *Li*_*1*_ (86.9%) fibers was substantially lower than its NIL, DP-5690 (94.5%). However, the reduction was not statistically significant (p> 0.05) due to high variations among the *Li*_*1*_ samples (Fig 3B). Average cellulose content of *Li*_*2*_ fibers (85.1%) was significantly (p<0.041) lower than its NIL, DP-5690 (Fig 3B).

### Chemical analyses of cotton fibers

#### Suberin and lignin depositions in the wild diploid *G*. *raimondii* fibers among the 1^st^ set of cotton materials

**Fig 4:**
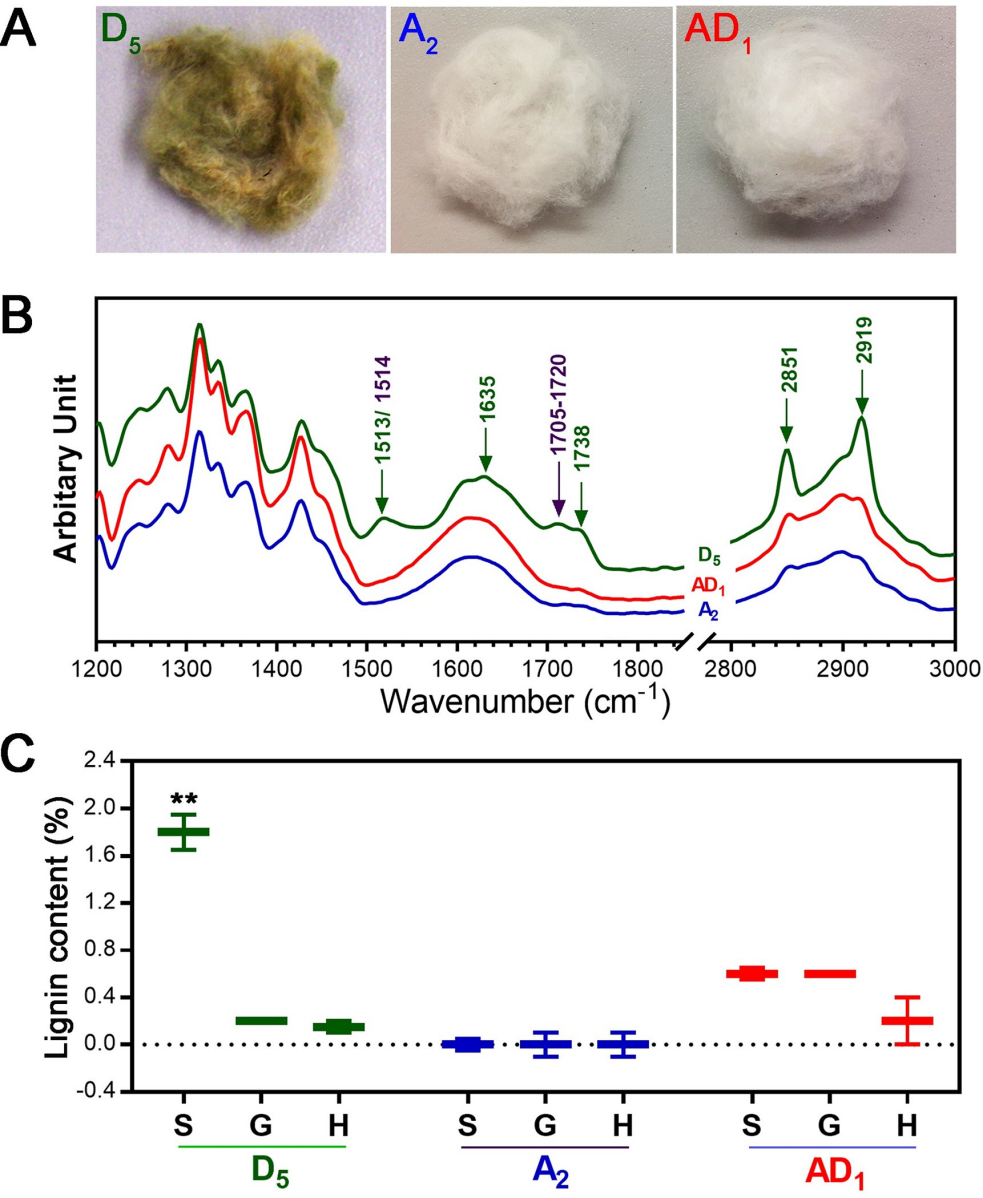
Suberin and lignin deposition in the wild diploid *G*. *raimondii* fibers among the 1^st^ set of cotton materials. A. Color differences of ginned and fully developed fibers from diploid *G*. *raimondii* D_5_-31 (D_5_), diploid *G*. *arboreum* A_2_-100 (A_2_), and polyploid *G*. *hirsutum* TM-1 (AD_1_). B. ATR FT-IR spectra. Each spectrum of the three cotton species fibers was normalized and compared. The wavenumbers representing suberin and lignin were labeled green and purple, respectively. C. Lignin quantification. Contents of syringyl (S), guaiacyl (G), and hydroxyphenyl (H) lignin were determined with pyrolysis-molecular beam mass spectrometry and compared among the three cotton species fibers. The error bars represent standard deviation. **p value < 0.01.

Among the three cotton species, wild *G*. *raimondii* produces naturally green colored fibers, whereas cultivated *G*. *arboreum* and *G*. *hirsutum* are white cotton (Fig 4A). Chemical fiber properties were monitored with ATR FT-IR spectroscopy in the 600–4000 cm^-1^ region (Fig 4B). Multiple IR spectral peaks known to be indicatives of suberin (1513, 1635, 1738, 2850, and 2920 cm^-1^) and lignin (1514 and 1705–1720 cm^-1^) components [37–42] were specifically identified from the wild *G*. *raimondii* fibers (S2 Table and Fig 4B).

Lignin levels were measured using a molecular beam mass spectrometer (Fig 4C). Among the three lignin units including 4-hydroxyphenyl (H), guaiacyl (G), and syringyl (S) units in the lignin polymer, the S lignin in *G*. *raimondii* fiber (1.8%) was significantly (p = 0.008) greater than cultivated *G*. *arboreum* (0%) and *G*. *hirsutum* (0.6%) fibers. In contrast, both G lignin (p = 0.18) and H lignin (p = 0.31) showed insignificant variation among the three cotton species.

#### Suberin and lignin depositions in the polyploid *G*. *hirsutum Li*_*1*_ and *Li*_*2*_ mutant fibers among the 2^nd^ set of cotton materials

**Fig 5:**
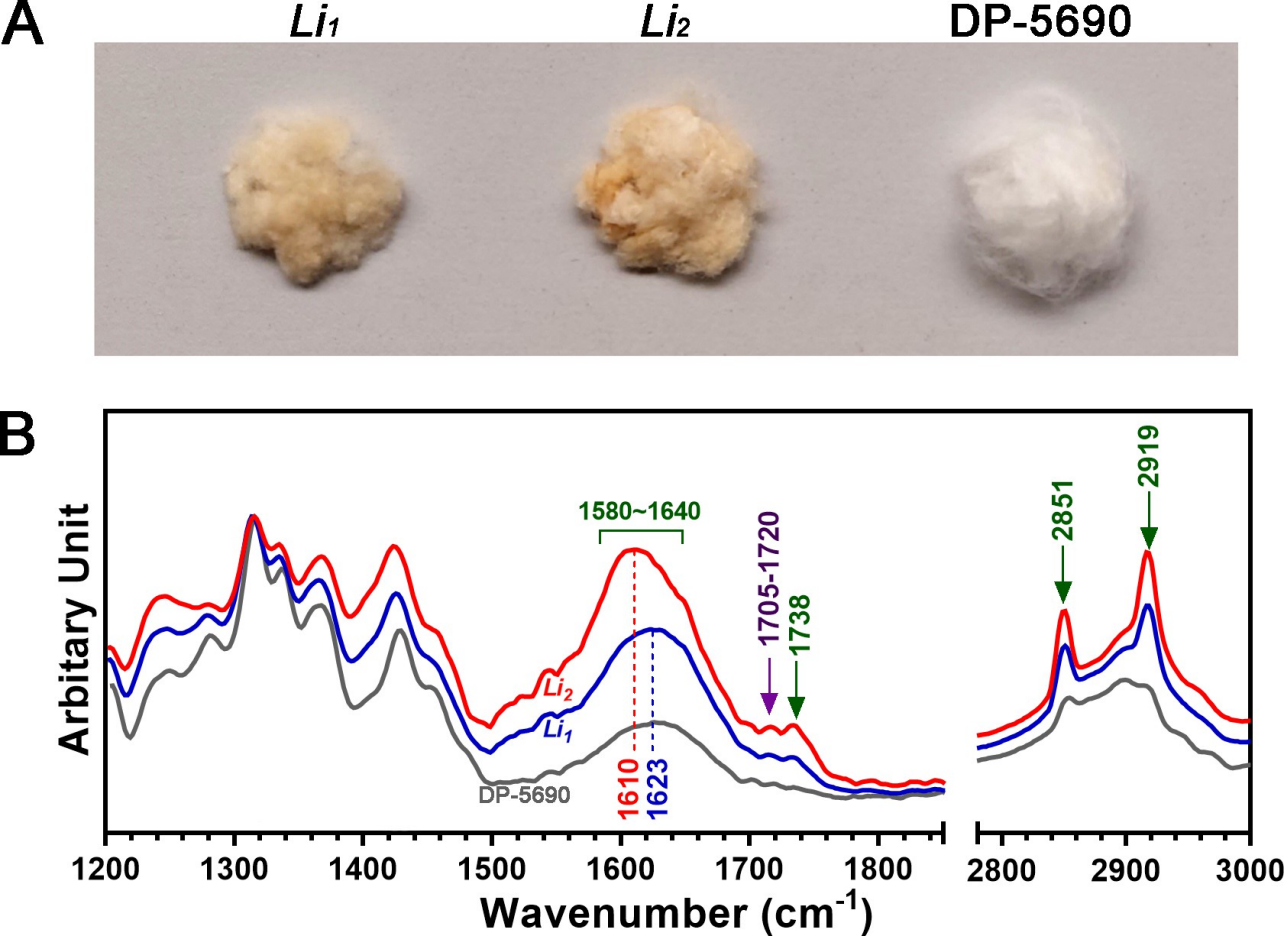
Suberin and lignin deposition in the polyploid *G*. *hirsutum Li*_*1*_ and *Li*_*2*_ mutant fibers among the 2^nd^ set of cotton materials. A. Color detection from the developed *Li*_*1*_ and *Li*_*2*_ mutant fibers. B. Normalized ATR FT-IR spectra. The wavenumbers representing suberin and lignin were described with green and purple fonts, respectively.

The unginned *Li*_*1*_ and *Li*_*2*_ fibers as well as their NIL fibers appear to be white (Fig 1B). On the contrary, the ginned *Li*_*1*_ and *Li*_*2*_ fibers showed a brown color that was visually different from their NIL fibers showing a white color (Fig 5A). Consistently, the IR spectra of *Li*_*1*_ and *Li*_*2*_ fibers were also different from those of DP-5690 fibers (Fig 5B). Multiple IR spectral peaks assigned as suberin (1738, 2850, and 2920 cm^-1^) and lignin (1705–1720 cm^-1^) components were specifically detected in the *Li*_*1*_ and *Li*_*2*_ short fibers (Fig 5B). In addition, a bigger bulge area (1580~1640 cm^-1^) of IR spectrum was detected from the short mutant fibers as compared with the NIL fibers. The bulge area may be composed of four close IR peaks (1588, 1606, 1624, and 1635 cm^-1^) that were assigned as suberin fractions from other plants [37, 41].

### Transcriptomic profiles of developing cotton fibers

#### Classification of developmental stages of the 1^st^ set of cotton fibers retrieved from the original RNA-seq analyses

As summarized in Table 1, RNA-seq data of developing *G*. *raimondii*, *G*. *arboreum*, and *G*. *hirsutum* fibers at 10, 20, or 28 DPAs were available from a public database. Because they are distinct species and grown in different environments [34–36], a closer examination of the RNA-seq data was needed to better align overall expression profiles and the fiber growth stages for more meaningful comparisons. The fiber developmental stages of the three cotton species used in the original research [34–36] were first classified by monitoring the transcript abundance and patterns of the indicator genes including *fasciclin-like arabinogalactan* [43] and *expansin* [44] that are specifically up-regulated at PCW stage as well as *cellulose synthase* (*CesA*) genes that are specifically up-regulated at SCW stage [45]. Three *Fasciclin-like arabinogalactan* genes and four *expansin* genes were commonly up-regulated in the 10 DPA fibers of all three cotton species (Table 2), suggesting that developing *G*. *raimondii*, *G*. *arboreum*, and *G*. *hirsutum* fibers at 10 DPA used in the original research were at the PCW stage. Three SCW *CesA* genes were up-regulated in developing *G*. *arboreum* and *G*. *raimondii* fibers at 20 DPA. In contrast, they were up-regulated in developing *G*. *hirsutum* TM-1 fiber at 28 DPA, but not at 20 DPA. The RNA-seq data of *G*. *raimondii* (20 DPA), *G*. *arboreum* (20 DPA), and *G*. *hirsutum* (28 DPA) were compared for studying SCW stage.

**Table 2:** Classifications of cotton fiber developmental stages of *G*. *raimondii*, *G*. *arboreum*, and *G*. *hirsutum*. Ten indicator genes are shown for each species according to genome type and at 10, 20, or 28 DPA. The most up-regulated RPKM numbers of each indicator genes in the cotton species were written in bold fonts.

Cotton ID	TAIR10 best hit	TAIR10 Description	*G*. *arboreum*		*G*. *raimondii*		*G*. *hirsutum*					
			A_2_-10	A_2_-20	D_5_-10	D_5_-20	A_T_-10	A_T_-20	A_T_-28	D_T_-10	D_T_-20	D_T_-28
Gorai.013G152900	AT5G55730	Fasciclin-like arabinogalactan	**1389.5**	382.3	**162.1**	30.5	**363.8**	324.6	22	25.6	**96.5**	22.2
Gorai.004G026800	AT3G55820	Fasciclin-like arabinogalactan	**358.7**	296.3	**423.9**	144.5	101.5	**134.2**	138.6	197.9	**244.4**	166.4
Gorai.006G147500	AT5G06390	Fasciclin like arabinogalactan	**2095.5**	1241.8	**949.6**	743.1	**10112.5**	7192.8	1952.2	**6126.4**	3972	1003.8
Gorai.001G083300	AT3G03220	expansin A13	**543.6**	153.8	**815.3**	175.1	**2086.8**	1086.1	93.8	**3404**	1772.1	139.7
Gorai.005G142200	AT2G03090	expansin A15	**279.7**	63.3	**942.7**	22.4	**583.7**	285.6	23.9	73.3	**141.5**	32.3
Gorai.003G131000	AT2G39700	expansin A4	**1078.2**	278.2	**4945.1**	46.8	**5431.3**	4293.2	0	**1912.7**	1579.2	0
Gorai.006G172400	AT2G39700	expansin A4	**367.7**	165.1	**1839.1**	30.5	363.8	**478.4**	4.8	1491.3	**2518.3**	9.7
Gorai.001G238100	AT5G44030	cellulose synthase A4	0	**1054**	9.3	**6427.3**	0	17.1	**21908.6**	0	22.5	**35749.3**
Gorai.004G057400	AT5G44030	cellulose synthase A4	76.7	**21670.8**	162.1	**66777.2**	188.9	202.6	**97586.7**	183.2	231.6	**61131.7**
Gorai.001G044700	AT5G17420	Cellulose synthase A7	20.3	**18033.8**	0	**41768.4**	5.6	0	**4996.5**	7.3	25.7	**84363.2**

#### Transcriptomic analyses of the 1^st^ set of cotton species at PCW or SCW stage

**Fig 6:**
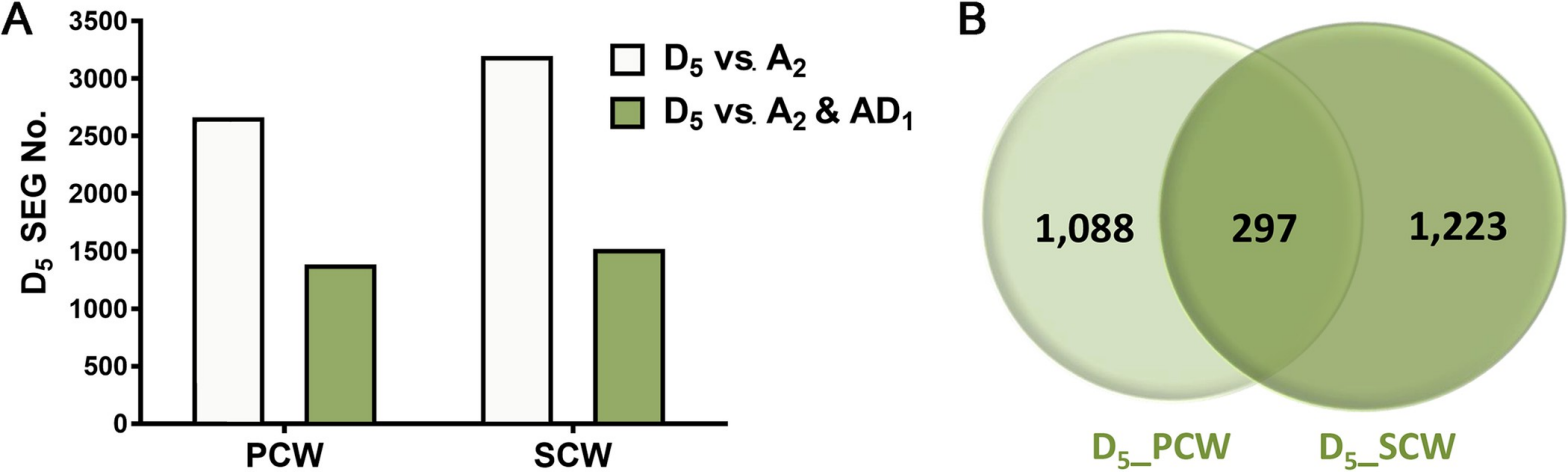
Transcriptomic analyses of wild diploid *G*. *raimondii* and two cultivated cotton species (1^st^ set). A. Quantification of specifically expressed genes (SEGs) in developing *G*. *raimondii* fibers at primary or secondary cell wall (PCW or SCW) stages by comparing them with *G*. *arboreum* (D_5_ vs A_2_) as well as *G*. *arboreum* and *G*. *hirsutum* (D_5_ vs A_2_ & AD_1_). B. Venn diagrams representing the common SEGs in *G*. *raimondii* fibers between PCW (D_5__PCW) and SCW (D_5__SCW) stages.

The expressed gene (EGs) numbers in *G*. *raimondii* D_5_ genome at PCW (18,698 EGs) and SCW (18,638 EGs) stages were similar to those in *G*. *hirsutum* D_T_ subgenome at PCW (15,485 EGs), and SCW (17,103 EGs) stages. The EG numbers in the *G*. *arboreum* A_2_ genome at PCW (18,333 EGs) and SCW (17,520 EGs) stages were also similar to those in the *G*. *hirsutum* A_T_ subgenome at PCW (16,188 EGs), and SCW (17,472 EGs) stage. In this study, the focus was on identification of genes specifically expressed in developing *G*. *raimondii* fibers, but not expressed (zero RPKM) in developing fibers of the other cotton species. Transcriptomic profiles between the two diploid cotton species identified specifically expressed genes (SEGs) in developing *G*. *raimondii* fibers at PCW (2,663 SEGs) and SCW (3,193 SEGs) stage as shown in Fig 6A. When the transcriptomic profiles of the three cotton species compared, 1,385 and 1,520 SEGs were identified from developing *G*. *raimondii* at PCW and SCW stages, respectively (Fig 6A and S3 Table). Between PCW and SCW stages of developing *G*. *raimondii* fibers, 297 SEGs were overlappingly expressed (Fig 6B).

Of the 297 SEGs, glycerol-3-phosphate acyltransferase (GPAT, Gorai.008G098000) is required for suberin biosynthesis in *Arabidopsis* (S4 Table) [46]. Peroxidase (Gorai.013G005800) related to lignin biosynthesis [47]. Laccases (Gorai.007G376600 and 004G234200) are involved in phenylpropanoid pathway producing suberin and lignin [47]. Cytochrome P450 family genes (Gorai.001G222700, 002G097500, 003G077000, 006G217000 and 010G166100) shown in S4 Table play important roles in detoxification of xenobiotics and stress responses [48]. Gene ontology enrichment analysis using Singular Enrichment Analysis of agriGO v. 2.0 [33] identified four GO categories including O-methyltransferase activity (GO:0008171), terpene synthesis activity (GO:0010333), tetrapyrrole binding (GO:0046906), and response to endogenous stimulus (GO:0009719) in developing *G*. *raimondii* fibers at PCW stage (Table 3). Three GO categories (O-methyltransferase activity, terpene synthesis activity, and tetrapyrrole binding) were also found at SCW stage. Multiple O-methyltransferases were reported to be involved in redundant functions for lignin, suberin and flavonoids [49]. Terpene synthesis proteins also control synthesis of secondary metabolites and gossypol biosynthesis by responding to various abiotic stresses [12]. Tetrapyrroles are required for detoxification of reactive oxygen species (ROS), programed cell death, photosynthesis and respiration [50]. Consistent with the previous report [13], GO (GO:0009719) involved in disease resistance was also over-represented in *G*. *raimondii* fibers (Table 3 and S3 Table).

**Table 3:** GO enrichment analyses of specifically expressed genes in PCW or SCW stage of developing *G*. *raimondii* (D_5_) fibers. List and annotation of genes are described in S3 Table.

GO term	Common mechanism	D_5__PCW	D_5__SCW
GO:0008171	O-methyltransferase activity	12 genes (p<0.0001)	10 genes (p<0.0001)
GO:0010333	Terpene synthesis activity	8 genes (p<0.0001)	16 genes (p<0.0001)
GO:0046906	Tetrapyrrole binding	27 genes (p<0.0001)	44 genes (p<0.0001)
GO:0009719	Response to endogenous stimulus	10 genes (p<0.01)	

#### Transcriptomic analyses of the 2^nd^ set consisting of *G*. *hirsutum* NILs differing in fiber length

**Fig 7:**
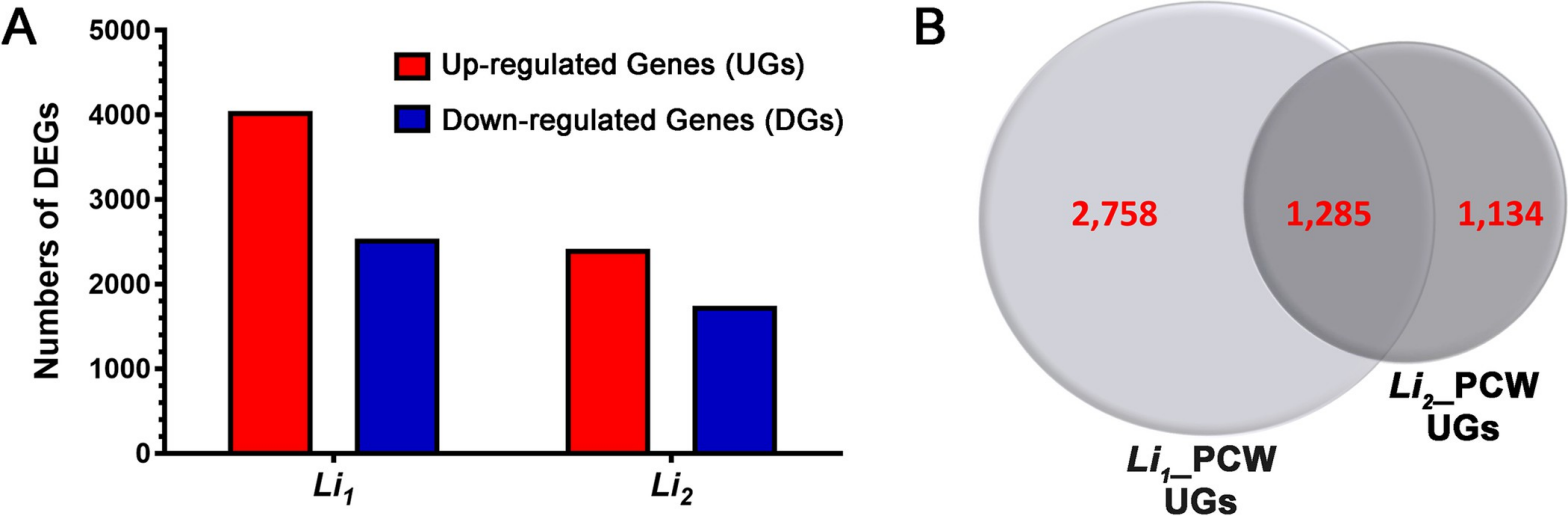
Transcriptomic analyses among three *G*. *hirsutum* NIL fibers differing in length. A. Summary of up- or down-regulated DEGs of *G*. *hirsutum* short fiber *Li*_*1*_ and *Li*_*2*_ mutant fibers at PCW stage with the corresponding NIL DP-5690 fiber. The DEGs annotated with JGI *Gossypium raimondii* reference genome were listed in S5 Table. B. Venn diagrams of the up-regulated genes (UGs) in developing *G*. *hirsutum Li*_*1*_ and *Li*_*2*_ mutant fibers.

The original RNA-seq analysis of the short fiber mutants (*Li*_*1*_ and *Li*_*2*_) was performed with total RNAs extracted from developing fibers at PCW stage (8–12 DPA) grown in greenhouse or cotton fields [22, 24]. To identify differentially expressed genes (DEGs), developing fibers from field grown *Li*_*1*_ and *Li*_*2*_ fibers were compared to their NIL, *G*. *hirsutum* DP-5690, using a 2-fold difference as a threshold. In the *Li*_*1*_ mutant fibers, 4,043 genes were up-regulated whereas 2,536 genes were down-regulated (Fig 7A). In the *Li*_*2*_ mutant fibers, 2,419 genes were up-regulated, whereas 1,740 genes were down-regulated (Fig 7A). Identification of candidate genes producing the color pigments in the two mutant fibers focused on the 1,285 genes (S5 Table) that were commonly up-regulated in the developing *Li*_*1*_ and *Li*_*2*_ fibers (Fig 7B).

GO enrichment analysis of the 1,285 UGs identified six GO categories (Table 4). The two GO categories including transporter activity (GO:0005215) and cellular respiration (GO:0045333) were also previously identified in the original analysis [24] by MapMan ontology [51]. Among the newly identified four GO categories, ADP binding (GO:0043531) and protein kinase activity (GO:0004672) composed of multiple nucleotide-binding leucine-rich repeat receptors (NLRs) and leucine-rich repeats receptor-like kinase (LRR-RLK) are involved in plant development and stress responses in other plants [52]. The other two GO categories, tetrapyrrole binding (GO:0046906) and response to endogenous stimulus (GO:0009719), were also over-represented in wild diploid *G*. *raimondii* fibers (Tables 3 and 4).

**Table 4:** GO enrichment analyses of up-regulated genes in *G*. *hirsutum Li*_*1*_ and *Li*_*2*_ short fibers at PCW stage. List and annotation of genes are described in S5 Table.

GO term	Description	Number of genes	p-value
GO:0005215	transporter activity	50	0.00065
GO:0045333	cellular respiration	7	0.00095
GO:0043531	ADP binding	28	9.5e-14
GO:0004672	protein kinase activity	75	1.2e-06
GO:0046906	tetrapyrrole binding	30	3.4e-07
GO:0009719	response to endogenous stimulus	9	7.6e-05

### Integration of the chemical phenotypes and transcriptomic profiles between the two sets of cotton materials differing in fiber lengths and cellulose contents

**Fig 8:**
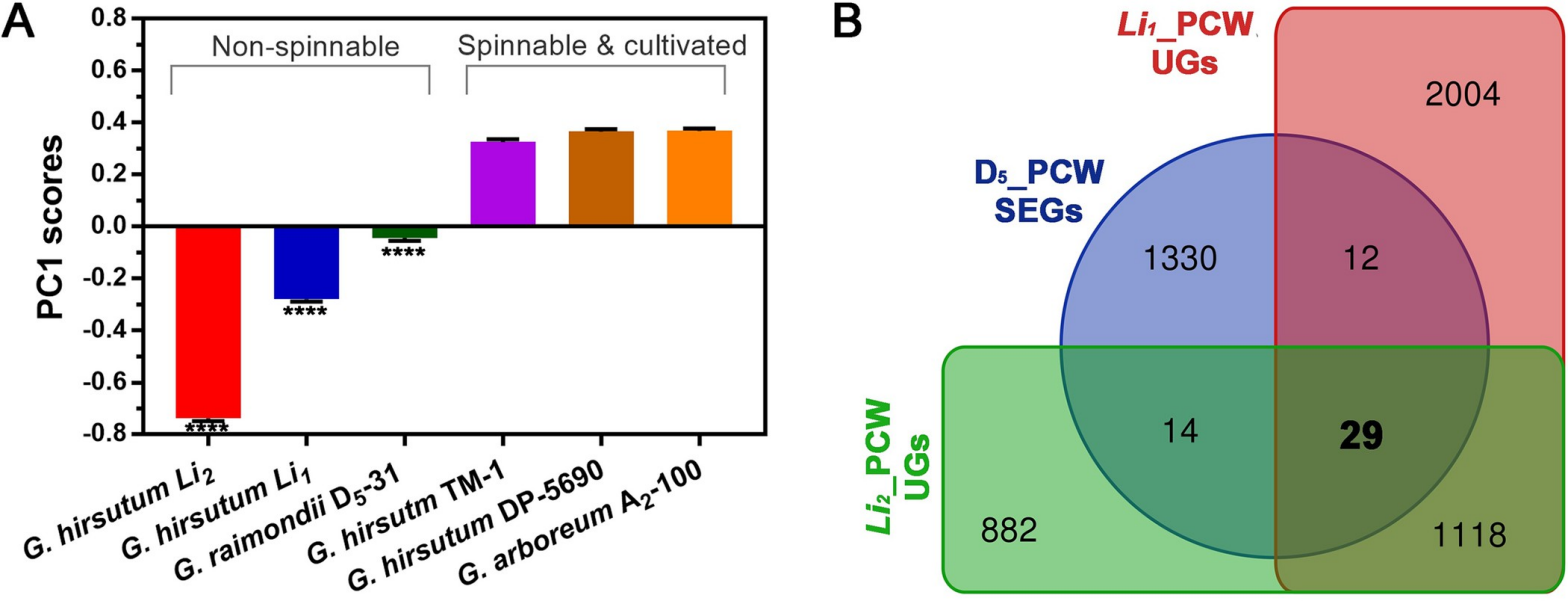
Integrations of chemical phenotypes and transcriptomic profiles between the two sets of cotton materials differing in fiber lengths and cellulose content. A. Classification of the six cotton samples from the first principal component (PC1) scores of principal component analysis operation on normalized ATR FT-IR spectra. B. Venn diagrams comparing the *G*. *raimondii* specifically expressed genes (SEGs) at PCW stage with the *G*. *hirsutum Li*_*1*_ and *Li*_*2*_ up-regulated genes (UGs) at PCW stage. The error bars represent standard deviation. ****p value *<*0.0001.

For an examination of the quantitative and statistical significances of the spectral features showing different chemical components among the fiber samples used in the 1^st^ and 2^nd^ sets, a principal component analysis (PCA) was performed with the spectral region (1200–3000 cm^-1^) composed of IR peak bands of suberin, lignin, and cellulose (Fig 8A). The analysis showed a dominant first principal component (PC1) accounting for 75.9% of the total variation, and revealed a distinction in PC1 score within the six tested samples (Fig 8A). The PC1 score increased in the order of *G*. *hirsutum Li*_*2*_ < *G*. *hirsutum Li*_*1*_ < *G*. *raimondii* D_5_-31 < *G*. *hirsutum* TM-1 ≈ *G*. *hirsutum* DP-5690 ≈ *G*. *arboreum* A_2_-100. The three cultivated cottons of *G*. *arboreum* A_2_-100 (0.369), *G*. *hirsutum* DP-5690 (0.365), and *G*. *hirsutum* TM-1 (0.326) demonstrated similar positive PC1 scores with insignificant (p = 0.587) variations. In contrast, the other three short fiber cottons of *G*. *hirsutum Li*_*2*_ (-0.738), *G*. *hirsutum Li*_*1*_ (-0.279), and *G*. *raimondii* D_5_-31 (-0.043) had all negative scores that were significantly (p<0.0001) variable from the cultivated cotton species. Among the three short fiber cottons, the PC1 scores also showed significant (p<0.0001) variations. These results suggested that the chemical compositions of *G*. *raimondii* D_5_-31, *G*. *hirsutum Li*_*1*_, and *G*. *hirsutum Li*_*2*_ were different despite a sharing of suberin and lignin fractions in addition to cellulose.

Of the UGs in *G*. *hirsutum Li*_*1*_ (4,043) and *Li*_*2*_ (2,419) fibers (Fig 7), there were redundant and homeologous genes in polyploid *G*. *hirsutum Li*_*1*_ (880) and *Li*_*2*_ (376) mutants. To identify the commonly up-regulated orthologous genes between the diploid D_5_ genome and polyploid AD_1_ genome composed of A_T_ and D_T_ subgenomes, we compared the diploid *G*. *raimondii* 1,385 SEGs with non-redundant UGs in polyploid *G*. *hirsutum Li*_*1*_ (3,163) and *Li*_*2*_ (2,043) (Fig 8B). Among the three short cottons, 29 orthologs were commonly up-regulated (Table 5). Consistent with the co-existence of lignin and suberin components in the three short cottons, a laccase (Gorai.009G260600_D_T_) and a peroxidase (Gorai.013G005800_A_T_) involved in lignin polymerization by oxidizing lignin monomers (monolignols) [47, 53, 54] as well as ABC-2 type transporter family gene (Gorai.002G062500_D_T_) required for suberin biosynthesis in *Arabidopsis* [55] were found. Jasmonate-zim-domain protein 8 (JAZ8, Gorai.009G154300) involved in flavonoid synthesis [56] was also found. A protein kinase protein (Gorai.011G182700) responsible for tetrapyrrole metabolism in a *Arabidopsis* color mutant was also up-regulated [57]. The other annotated genes including Fe(II)/ascorbate oxidase (SRG1) [58], cysteine-rich receptor-like protein kinase 10 (CRK10) [59], leucine-rich repeat protein kinase [60], FAD-binding berberine family protein [61], glutathione S-transferase [62], PLAT/LH2 domain-containing lipoxygenase [63], FLOTILIN2 (FLOT2) [64], cytokinin response activator (ARR1) [65], PSBP-domain protein 6 (PPD6) [61], cyclic nucleotide gated channel 1 (CNGC) [66], and NAC014 [67] were all related to immunity or stress responses. Seven of them have not been annotated and the 22 annotated genes were not sufficient to perform the GO enrichment analysis.

**Table 5:** Commonly up-regulated orthologous genes among developing *G*. *raimondii* D_5_, *G*. *hirsutum Li*_*1*_ and *Li*_*2*_ fibers at PCW stage.

No	*G*. *raimondii*		*G*. *hirsutum*			Description
	D_5_ gene ID	RPKM*	AD_1_ gene ID	*Li*_*1*_ /DP-5690^§^	*Li*_*2*_ /DP-5690^§^	
1	Gorai.010G058200	145.9	Gorai.010G058200_A_T_	3.0	19.7	Fe(II)/ascorbate oxidase gene SRG1
2	Gorai.013G005800	132.0	Gorai.013G005800_A_T_	17.2	16.0	Peroxidase superfamily protein
3	Gorai.002G062500	99.6	Gorai.002G062500_D_T_	3.7	7.0	ABC-2 type transporter family protein
4	Gorai.001G266300	39.4	Gorai.001G266300_D_T_	2.1	2.1	Lysine ketoglutarate reductase trans-splicing protein
5	Gorai.011G017700	37.1	Gorai.011G017700_A_T_	2.3	2.1	CRK10 (cysteine-rich receptor-like protein kinase 10)
6	Gorai.007G338100	27.8	Gorai.007G338100_A_T_	11.3	2.8	Disease resistance LRR family protein
7	Gorai.009G389300	25.5	Gorai.009G389300_D_T_	13.9	6.1	Protein of unknown function (DUF2921)
8	Gorai.005G023800	20.9	Gorai.005G023800_A_T_	9.2	59.7	FAD-binding Berberine family protein
			Gorai.005G023800_D_T_	6.1	45.3	
9	Gorai.009G154300	13.9	Gorai.009G154300_A_T_	4.6	4.6	Jasmonate-zim-domain protein 8 (JAZ8)
10	Gorai.005G013300	13.9	Gorai.005G013300_A_T_	14.9	3.7	NAC 014
11	Gorai.005G163000	13.9	Gorai.005G163000_A_T_	4.0	3.3	Lysine ketoglutarate reductase trans-splicing protein
12	Gorai.011G006900	7.0	Gorai.011G006900_D_T_	4.3	3.5	Leucine-rich repeat protein kinase family protein
13	Gorai.013G097700	7.0	Gorai.013G097700_D_T_	9.2	3.0	O-fucosyltransferase family protein
14	Gorai.011G163600	4.6	Gorai.011G163600_D_T_	13.0	8.6	Glutathione S-transferase tau 7
15	Gorai.001G228100	4.6	Gorai.001G228100_A_T_	2.1	5.7	PLAT/LH2 domain-containing lipoxygenase
16	Gorai.006G135600	4.6	Gorai.006G135600_D_T_	39.4	4.0	FLOTILIN2 (FLOT2)
17	Gorai.009G260600	2.3	Gorai.009G260600_D_T_	11.3	4.0	Laccase 14
18	Gorai.007G074700	2.3	Gorai.007G074700_A_T_	4.0	8.0	TPX2 (targeting protein for Xklp2) protein
19	Gorai.009G006200	2.3	Gorai.009G006200_A_T_	3.7	8.6	cytokinin response activator (ARR1)
20	Gorai.013G125600	2.3	Gorai.013G125600_A_T_	4.0	1.5	PSBP-DOMAIN PROTEIN 6 (PPD6) thylakoid lumenal protein
			Gorai.013G125600_D_T_	7.0	2.1	
21	Gorai.011G182700	2.3	Gorai.011G182700_A_T_	2.6	14.9	Protein kinase superfamily protein
22	Gorai.001G155300	2.3	Gorai.005G223900_A_T_	5.7	8.6	Cyclic nucleotide gated channel 1

*The RPKM values were detected in developing *G*. *raimondii* fibers, but not detected in developing *G*. *arboreum* and *G*. *hirsutum* fibers.

^§^ Fold difference

## Discussion

### Common characteristics of physical properties of cotton fibers from wild diploid *G*. *raimondii* and polyploid *G*. *hirsutum Li*_*1*_ and *Li*_*2*_ mutants

Physical properties of cultivated cotton fibers are generally assessed by a High Volume Instrument (HVI) which is defined by the International Cotton Advisory Committee as a standardized instrument for cotton fiber quality measurements [7]. Cotton fibers with a length less than 12.7 mm are classified as short fibers that reduce the quality of spun yarns. The cotton fibers produced by *G*. *raimondii* and *G*. *hirsutum Li*_*1*_ and *Li*_*2*_ mutants were too short to be measured by HVI. Thus, we manually measured the maximum fiber lengths of the wet and relaxed cotton fibers from the chalezel end of cottonseeds [27]. The maximum fiber length of *G*. *raimondii* D_5_-6 (11.7 mm) and D_5_-31 (10.1 mm) as well as *G*. *hirsutum Li*_*1*_ (2.6 mm) and *Li*_*2*_ (8.1 mm) mutants were significantly shorter than those of cultivated diploid cotton, *G*. *arboreum* SXY1 (28.0 mm) and A_2_-100 (30.1 mm) as well as cultivated polyploid cotton, *G*. *hirsutum* TM-1 (41.7 mm), SG-747 (40.1 mm) and DP-5690 (38.7 mm) as shown in Figs 2A and 3A. There are two different types of *G*. *hirsutum* fibers. Lint fibers differentiate from ovule epidermis on the day of anthesis and they grow approximately 25~35 mm based on HVI measurements. In contrast, linter or fuzz differentiate from the ovule epidermis around 5 to 10 DPA and they do not grow longer than 15 mm [68]. Wild diploid *G*. *raimondii* was often described as a lintless, non-fibered, or fiberless species [12, 69, 70]. The full names of the *Li*_*1*_ and *Li*_*2*_ mutants also contain “lintless” [17, 18]. However, the fiber initials of *G*. *raimondii* [8], *G*. *hirsutum Li*_*1*_ mutant [19], and *G*. *hirsutum Li*_*2*_ mutant [20] all differentiate on the day of anthesis. Thus, the short fibers produced from *G*. *raimondii* and *G*. *hirsutum Li*_*1*_ and *Li*_*2*_ mutants can be classified as lint fibers according to the definition described by Lang [68].

In this study, we showed that the three short cottons of wild *G*. *raimondii* and two *G*. *hirsutum* mutants produced fibers containing color pigments composed of lignin and suberin (Figs 4A and 5A). The green coloration of *G*. *raimondii* fibers was reported by Hutchinson and his colleagues in 1947 [69], but its color pigment was not further characterized. A recent study showed that NIR spectra of *G*. *raimondii* fibers were similar to those measured from naturally green colored *G*. *hirsutum* fibers [71]. Despite the extensive studies of *Li*_*1*_ and *Li*_*2*_ mutant fibers, the brown color pigments of the short fiber mutants (Fig 5A) have been unnoticed. Green color of the cotton fibers is faded to tan color when they are exposed to light [72]. The light brown color of the short mutant fibers might have been overlooked because their NIL, DP-5690, produces white lint fibers.

### Common chemical components among diploid *G*. *raimondii* and polyploid *G*. *hirsutum* mutants

Chemical analyses using cellulose assay, ATR FT-IR spectroscopy, and mass spectrometry consistently showed suberin and lignin components in the three short fibers. Average cellulose contents of *G*. *raimondii* (75.0~78.0%) and *G*. *hirsutum Li*_*1*_ and *Li*_*2*_ mutants (85.1~86.9%) fibers were lower than cultivated *G*. *arboreum* (95.6~100%) and *G*. *hirsutum* (95.8~98.0%) fibers (Figs 2B and 3B). Non-cellulosic components of the *G*. *raimondii* (22.0~25.0%) and *G*. *hirsutum Li*_*1*_ and *Li*_*2*_ (13.1~14.9%) fibers were substantially greater than the cultivated fibers (0~4.4%). These results were consistent with the previous reports showing different cellulose contents between green and white upland cotton [71] and functional divergence of cellulose synthase orthologs between wild *G*. *raimondii* and cultivated *G*. *arboreum* [45].

The three short cottons of *G*. *raimondii* and *G*. *hirsutum Li*_*1*_ and *Li*_*2*_ mutants demonstrated the signature IR spectral peaks of suberin and lignin (Figs 4B and 5B). Another type of short fiber mutant *li*_*y*_ also showed the signature IR spectral peaks of suberin [73]. In naturally green colored *G*. *hirsutum*, suberin layers were observed in the secondary cell wall in cotton fibers [72, 74], and a major lignin precursor and its derivatives were deposited in the suberin layers [75]. Suberin and lignin can be produced with common precursors, i.e. phenolic components [76]. In contrast to suberin consisting of phenolics and aromatic polymers, lignin is purely composed of poly-aromatic components [76]. Generally, lignin is derived from three phenylpropanoid monomers, the monolignols 4-coumaryl, coniferyl, and sinapyl alcohols, that produce the 4-hydroxyphenyl (H), guaiacyl (G), and syringyl (S) units in the polymer [77]. Our mass spectrometry lignin analysis showed significantly greater content of the S lignin in wild *G*. *raimondii* fiber (1.8%) than those of the other cultivated cotton fibers (0~ 0.6%). Recent studies suggest that lignin may play an important role in cotton fiber quality [78, 79].

The integrative PCA method of the two sets enabled classifying the six cotton fibers into two classes according to the PC1 scores (Fig 8A). All three cultivated cottons shared similar positive PC1 scores without any significant variation, whereas all three short cottons showed negative PC1 scores with significant and broad variation. During cotton fiber development, underdeveloped cotton fibers containing high levels of non-cellulosic components show negative PC1 scores [80]. As cellulose content increases during normal fiber development, PC1 scores also increase and become positive [81]. Notably, the pattern of the PC1 score in Fig 8A was consistent with previous reports that PC1 scores increased with the cellulose content during cotton fiber development [45]. The IR bulge area likely represents a macromolecule complex composed of suberized components whose IR signals can overlap. Interestingly, there were noticeable IR peak bands of the bulge area among *G*. *hirsutum Li*_*1*_ (1623 cm^-1^), *G*. *hirsutum Li*_*2*_ (1610 cm^-1^), and *G*. *raimondii* D5 (1635 cm^-1^) (Figs 4B and 5B). These results along with the significant variation of their PC1 scores showed variation in the three short cottons although they shared suberin and lignin components (Figs 4B, 5B and 8A).

### Commonly up-regulated orthologs among diploid *G*. *raimondii* and polyploid *G*. *hirsutum* mutants

To test if suberin and lignin genes were specifically up-regulated in developing *G*. *raimondii* fibers, we used the RNA-seq data performed with the RNAs extracted from developing fibers of *G*. *raimondii*, *G*. *arboreum*, and *G*. *hirsutum* (Table 1) [34–36]. The original transcriptomic analyses of the short fiber mutants (*Li*_*1*_ and *Li*_*2*_) and their NIL DP-5690 were only performed with total RNAs extracted from developing fibers at PCW stage [22, 24]. Thus, we verified that suberin and lignin were specifically detected at the PCW stage of developing mutant fibers (S1 Fig). The short fiber phenotypes of *G*. *raimondii* fibers [70, 71] and mutants [19, 20, 22, 24] were consistent across various growing conditions. In this study, we used the JGI *G*. *raimondii* D_5_ reference genome for analyzing transcriptomic profiles of the two sets because the *G*. *raimondii* genome sequence shows high homology (>96%) with the coding sequences of *G*. *hirsutum* A_T_ and D_T_ subgenomes. Thus, the D_5_ reference genome sequence has been successfully used for characterizing the *Li*_*1*_ and *Li*_*2*_ genomes by several groups [19, 20, 22, 24, 82]. Transcriptomic analysis of the 1^st^ set identified that genes involved in suberin and lignin biosynthesis were specifically expressed in *G*. *raimondii* fibers (Fig 6, Table 3 and S3 Table). Among them, glycerol-3-phosphate acyltransferase 1 (GPAT), cytochrome P450 family genes, and laccases were reported to be involved in suberin or lignin biosynthesis in other plants [46, 47, 53, 54, 76]. Among the four GO categories over-represented in *G*. *raimondii* fibers (Table 3), O-methyltransferase activity is essential for biosynthesis of lignin, suberin and flavonoids [49, 83]. Integrative analyses of the two sets identified 29 genes that were commonly up-regulated in wild cotton species and short fiber mutant fibers (Table 5). Of the 22 annotated genes, four genes such as laccase, peroxidase, ABC-2 type transporter, and JAZ8 are involved in biosynthetic processes of lignin, suberin, and their derivatives [47, 54–56, 84]. The other 18 annotated genes are reported to be related to stress responses (Table 5). The mutations of an *actin* [25] and a putative *Ran Binding Protein 1* [23] cause the short fiber phenotypes of the *Li*_*1*_ and *Li*_*2*_ mutant respectively, and also up-regulate the genes involved in stress responses including lignin, suberin and flavonoid biosynthesis (Table 5).

Lignin deposition was suggested to reduce the extensibility of expanding fiber cell walls [79]. Suberin has been reported to be a major regulator of water and solute transport, and a pathogen barrier in plant cell walls [76]. A recent functional study of *Arabidopsis* mutants altered in suberin deposition clearly showed the reductions of the apoplastic transport of water and ions [85]. Hydrophobic suberin in the cotton fiber cell walls also negatively affect apoplastic transport activities in cotton fibers [72, 74, 86].

### Conclusion

Here, we used both phenotypic and transcriptomic analyses for identifying common mechanisms reducing fiber elongation in the short fibers generated from *G*. *hirsutum Li*_*1*_ and *Li*_*2*_ mutants as well as wild *G*. *raimondii*. Chemical analyses identified a common deposition of suberin and lignin in the short fiber cell walls. The genes involved in suberin and lignin biosynthesis were also commonly up-regulated in the elongating cotton fibers of the three short cottons as compared with the cultivated and long *G*. *arboreum* and *G*. *hirsutum* fibers. These results support a notion that suberin and lignin deposition may affect cotton fiber elongation process negatively. They also provide insight on how suberin and lignin biosynthesis can affect fiber length and cellulose productions in wild and cultivated cotton species.

## Supporting information

S1 Fig Suberin and lignin deposition in developing *G*. *hirsutum Li*_*2*_ mutant fiber at various developmental stages. (DOCX)

S1 Table Detailed information of the selected RNA-seq experiments in public database. (XLSX)

S2 Table Characteristic ATR-FTIR spectral peaks of suberins and lignins in cotton fibers. (DOCX)

S3 Table Annotation of specifically expressed genes in developing *G*. *raimondii* fibers at primary and secondary wall biosynthesis stages as compared with developing *G*. *arboreum* and *G*. *hirsutum* fibers. (XLSX)

S4 Table List of genes potentially involved in suberin and lignin biosynthesis in *G*. *raimondii* fibers. (XLSX)

S5 Table Annotation of differentially expressed genes in developing *G*. *hirsutum Li*_*1*_ and *Li*_*2*_ mutant fibers at primary wall biosynthesis stage as compared with their near isogenic line, *G*. *hirsutum* DP-5690 fibers. (XLSX)
