# Supplementary material for: Phenomics and transcriptomics analyses reveal deposition of suberin and lignin in the short fiber cell walls produced from a wild cotton species and two mutants
Source: PLoS One. 2023 Mar 9;18(3):e0282799. doi: 10.1371/journal.pone.0282799 (PMC9997941; doi:10.1371/journal.pone.0282799)
Supplement: S1 Fig — (DOCX) [file pone.0282799.s001.docx]

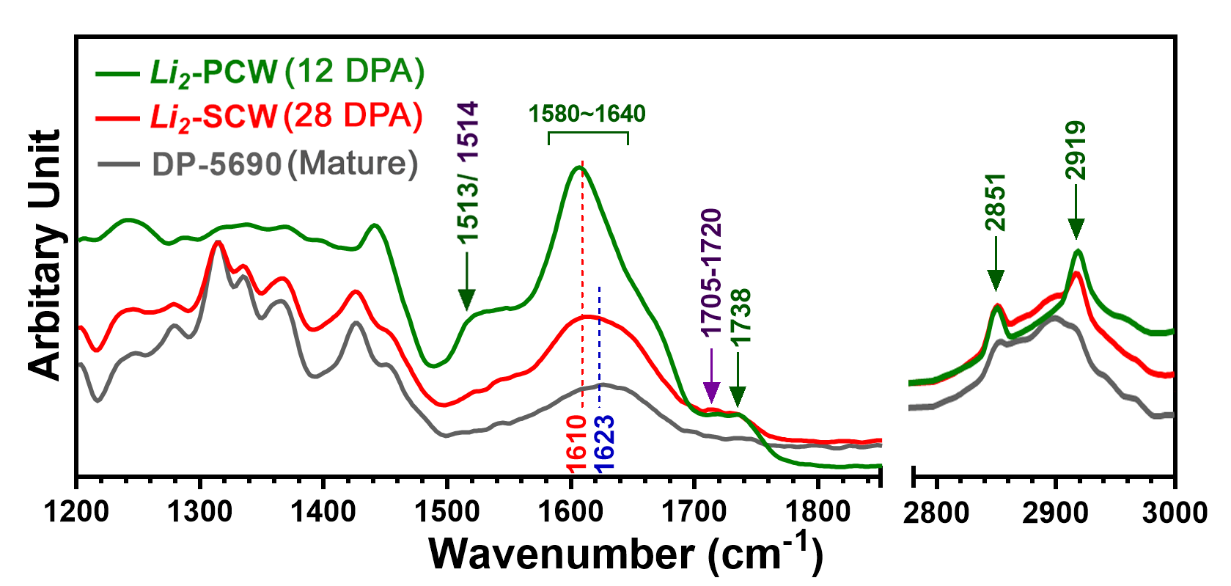


**S1 Fig. Suberin and lignin deposition in developing *G. hirsutum* *Li_2_*** **mutant fibers at various developmental stages**. Developing *Li_2_* mutant fibers at primary cell wall stage (*Li_2_*-PCW, 12 DPA) and secondary cell wall stage (*Li_2_*-SCW, 28 DPA) and fully developed DP-5690 fibers (DP-5690 Mature, 48 DPA) were harvested from field grown NIL cotton plants. ATR FT-IR spectra of the three cotton fiber samples was measured, normalized and analyzed. The IR spectra representing suberin (1513, 1580-1640, 1738, 2851, and 2919 cm^-1^) and lignin (1514 and 1705-1720 cm^-1^) identified from the fully developed *G. raimondii* and *G. hirsutum* *Li_1_* and *Li_2_* mutant fibers (Fig. 4 and 5) were also detected specifically from the developing *Li_2_* mutant fibers at PCW and SCW stages.
