# Supplementary material for: Phenomics and transcriptomics analyses reveal deposition of suberin and lignin in the short fiber cell walls produced from a wild cotton species and two mutants
Source: PLoS One. 2023 Mar 9;18(3):e0282799. doi: 10.1371/journal.pone.0282799 (PMC9997941; doi:10.1371/journal.pone.0282799)
Supplement: S2 Table — (DOCX) [file pone.0282799.s003.docx]

**S2 Table. Characteristic ATR-FTIR spectral peaks of suberins and lignins in cotton fibers**

| **IR peak**  **(cm^-1^)** | **Cell wall component** | **Vibration mode** | **Reference** |
| --- | --- | --- | --- |
| 1513/1514 | Suberin / lignin | Aromatic skeleton | [36, 37] |
| 1588 | Suberized fractions | C-C aromatic | [40] |
| 1606 | Suberin fractions | C-C aromatic | [40] |
| 1624 | Suberin fractions | C=C phenolic acid | [40] |
| 1635 | Suberin | C=C double bonds | [36] |
| 1705-1720 | Lignin | Carbonly/carboxyl stretch | [37] |
| 1738 | Suberin / Fatty acid ester | Acetyl group C=O | [36, 41] |
| 2851 | Suberin | Aliphatic C-H | [36, 38] |
| 2919 | Suberin | Aliphatic C-H | [36, 38] |

36. Cordeiro N, Belgacem M, Silvestre A, Neto CP, Gandini A. Cork suberin as a new source of chemicals.: 1. Isolation and chemical characterization of its composition. International Journal of Biological Macromolecules. 1998;22(2):71-80.

37. Boeriu CG, Bravo D, Gosselink RJ, van Dam JE. Characterisation of structure-dependent functional properties of lignin with infrared spectroscopy. Industrial crops and products. 2004;20(2):205-18.

38. Socrates G. Infrared and Raman characteristic group frequencies: tables and charts: John Wiley & Sons; 2004.

39. Boza Barducci T, Madoo RM. Investigaciones acerca del parentesco de la especie peruana de algodonero Gossypium raimondii, Ulbrich. Estacion Experimental Agricola de la Molina, Lima, Peru. Boletin. 1941;(22).

40. Järvinen R. Cuticular and suberin polymers of edible plants. Analysis by gas chromatographic-mass spectrometric and solid state spectroscopic methods [Dissertation]: University of Turku; 2010.

41. Stewart D. Fourier transform infrared microspectroscopy of plant tissues. Applied Spectroscopy. 1996;50(3):357-65.
